# Effectiveness of Upper Limb Wearable Technology for Improving Activity and Participation in Adult Stroke Survivors: Systematic Review

**DOI:** 10.2196/15981

**Published:** 2020-01-08

**Authors:** Jack Parker, Lauren Powell, Susan Mawson

**Affiliations:** 1 University of Derby Derby United Kingdom; 2 University of Sheffield Sheffield United Kingdom

**Keywords:** wearable electronic devices, stroke, rehabilitation, upper extremity

## Abstract

**Background:**

With advances in technology, the adoption of wearable devices has become a viable adjunct in poststroke rehabilitation. Upper limb (UL) impairment affects up to 77% of stroke survivors impacting on their ability to carry out everyday activities. However, despite an increase in research exploring these devices for UL rehabilitation, little is known of their effectiveness.

**Objective:**

This review aimed to assess the effectiveness of UL wearable technology for improving activity and participation in adult stroke survivors.

**Methods:**

Randomized controlled trials (RCTs) and randomized comparable trials of UL wearable technology for poststroke rehabilitation were included. Primary outcome measures were validated measures of activity and participation as defined by the International Classification of Functioning, Disability, and Health. Databases searched were MEDLINE, Web of Science (Core collection), CINAHL, and the Cochrane Library. The Cochrane Risk of Bias Tool was used to assess the methodological quality of the RCTs and the Downs and Black Instrument for the quality of non RCTs.

**Results:**

In the review, we included 11 studies with collectively 354 participants at baseline and 323 participants at final follow-up including control groups and participants poststroke. Participants’ stroke type and severity varied. Only 1 study found significant between-group differences for systems functioning and activity (*P*≤.02). The 11 included studies in this review had small sample sizes ranging from 5 to 99 participants at an average (mean) age of 57 years.

**Conclusions:**

This review has highlighted a number of reasons for insignificant findings in this area including low sample sizes and the appropriateness of the methodology for complex interventions. However, technology has the potential to measure outcomes, provide feedback, and engage users outside of clinical sessions. This could provide a platform for motivating stroke survivors to carry out more rehabilitation in the absence of a therapist, which could maximize recovery.

**Trial Registration:**

PROSPERO CRD42017057715; https://www.crd.york.ac.uk/prospero/display_record.php?RecordID=57715

## Introduction

### Background

Stroke is a leading cause of mortality and disability worldwide [1], and the economic costs of treatment and poststroke care result in a mean cost to the economy of £46,039 a year per patient for the first 5 years after admission in England, Wales, and Northern Ireland alone [2]. Following a stroke, survivors are left with multiple impairments, and as a result only 5% to 20% will regain full function of the upper limb (UL) with up to 66% still being impaired in the chronic phase [3]. This often results in functional limitations in activities of daily living and decreased quality of life [4].

Over recent years, there has been a contextual shift in service delivery from hospital-based rehabilitation to the community. Although it has been recommended that rehabilitation should continue until maximum recovery has been achieved [5], owing to the increasing demand on services and financial constraints, service needs cannot be met; therefore, radical innovation and the adoption of a self-management paradigm are considered as a way of delivering independent home-based rehabilitation, thereby meeting the challenges faced in health care [6].

Evidence exists supporting the need for intensity and repetition of motor skills to promote neuroplasticity and motor relearning. With significant advances in information and communication technology (ICT) and more specifically in the rapid development and deployment of sensor technology for health care monitoring, a number of technological aids with a potential to measure and monitor poststroke activity have been explored for both the lower limb [7] and the UL [8]. However, many include the use of expensive, large, complex, ungainly equipment that is impractical to use in everyday contexts [9]. Therefore inexpensive, wearable, and commercially available sensors have become a more viable option for quantifying movements and activities during poststroke rehabilitation [10-12].

A number of recent systematic and nonsystematic reviews highlight the growing use of wearable devices to provide poststroke rehabilitation in both clinical and nonclinical settings for motion analysis and physical activity monitoring [12-17]. These include microelectromechanical systems containing accelerometers, gyroscopes, and magnetometers; fabric and body-worn sensor networks [18], pressure sensors [19-22], and physiological monitoring such as blood pressure and oxygen saturation [23,24]. Other wearable devices specifically designed and used for poststroke rehabilitation also include robotics [25], virtual reality [26], functional electrical stimulation (FES) [27,28], electromyographic biofeedback [29], and transcutaneous electrical nerve stimulation [30-32].

However, while these devices have the potential to reliably measure duration, frequency, intensity, and quality of activity and movement, all of which are key variables for poststroke recovery [33], no reviews have synthesized the evidence underpinning the use of these devices for independent poststroke UL rehabilitation. Therefore, the aim of this review will be to explore and examine how effective these medical devices are as interventions for improving the function of the UL in adult stroke survivors.

The International Classification of Functioning, Disability, and Health (ICF) [34] considers the interaction between pathology (body structure and function), impairment (signs and symptoms), activities (functionality), and participation (social integration), and it has now become the main conceptual framework for poststroke rehabilitation [5,35,36].

### Objective

For this review, we focused on the activities and participation domain of the ICF as this would provide an indication of how the interventions have or have not led to functional gains in everyday life.

## Methods

The review protocol was registered on PROSPERO (CRD42017057715). The review was undertaken in accordance with the general principles recommended in the Preferred Reporting Items for Systematic Reviews and Meta-Analyses [37].

### Definitions

Wearable technologies can be subdivided into those operating independently and functioning as central connectors for other devices “and” or “or” information (eg, wrist-worn fitness tracker and smartphone) and those capturing specific actions or executing a measurement (eg, heart rate monitor worn around the chest) offloading to a primary wearable device for analysis [38]. We define a wearable device in the context of poststroke rehabilitation as “a wearable device that is worn externally on the body that is portable (the user is able to wear the device but is free to move around and not fixed to a station) and is able to use the device independently of a therapist.”

### Search Methods

As per the Cochrane Handbook [39], the Population Intervention Comparison Outcome Study Design framework helped authors to define the inclusion and exclusion criteria and the search terms of this review. For this review, the population refers to poststroke adults, the intervention to technological interventions for UL rehabilitation in stroke survivors, and included studies included a comparison group and were not limited to randomized controlled trials (RCTs). The outcome focused on activity and participation measures of UL function poststroke. Search terms and databases were selected based on Cochrane literature and institutional information specialist advice.

The following databases were searched from the year 2000 to April 2019: MEDLINE, Web of Science (Core collection), CINAHL, Scopus, and the Cochrane Library. Medical Subject Headings (MeSH) keywords used were cerebrovascular disorders, hemorrhage, cerebral hemorrhage, sensory feedback, motor skills, physical therapy modalities, physical and rehabilitation medicine, exercise, exercise therapy, rehabilitation, exercise movement techniques, information technology, technology, self-help devices, telemedicine, upper extremity, arm, hand joints, shoulder joint, elbow joint, and wrist joint. Text terms used were stroke, UL, rehabilitation, and technology. These were combined with the following synonyms: CVA, cerebrovascular accident, poststroke, cerebrovascular, brain ischemia, brain vascular, upper extremity, arm, shoulder, hand, axilla, axilla, elbow, forearm, finger, wrist, physiotherapy, physical therapy, physiatric, exercise, biofeedback, sensory feedback, advise, train, therapy, treat, motor skills, motor relearn, re-educate, recovery, enhance, promote, support, function, activity, physical, information technology, IT, ICT, information and communications technology, assistive technology, telehealth, telecare, telerehabilitation, and wear. Boolean logic was used to combine terms using *AND* and *OR*. MeSH terms refer to specific terms that are recognized for indexing journals and books in electronic databases. The free text terms and synonyms were words used in the search strategy that was looked for in titles and abstracts. The MEDLINE search strategy can be found in Multimedia Appendix 1. Electronic citations were downloaded into a reference manager. The inclusion and exclusion criteria for the search strategy are presented in Textboxes 1 and 2, respectively.

As technology is changing very quickly, authors deemed technology before the year 2000 to be particularly outdated. RCTs and randomized comparable trials were chosen as the appropriate study design for inclusion in this review as the review aims to assess the effectiveness of the included interventions. Non-RCT and nonrandomized comparable trial evidence is therefore outside the scope of this review. Comparators (control groups) could include treatment as usual and exercise therapies that do not include any other intervention or sham stimulation.

The primary outcome measures for this review are those that assess activity or participation as defined by the World Health Organization (WHO) ICF [40]. These measures include the following: the Box and Blocks Test (BBT) [41]; Action Research Arm Test (ARAT) [42]; Barthel Index (BI) [43]; Chedoke Arm and Hand Activity Inventory (CAHAI) [44-47]; Jebson-Taylor Hand Function Test (JTHFT) [48]; Wolf Motor Function Test (WMFT) [49]; Motor Activity Log (MAL) [50]; Motor Assessment Scale (MAS) [51]; Stroke Impact Scale (SIS) [52]; the Rivermead Motor Assessment (RMA) [53]; Upper Extremity Function Test (UEFT) [54]; and the short version of disabilities of arm, shoulder, and hand (QuickDASH) [55].

A total of 3 measures of *system functioning* WHO ICF, namely, the Fugl-Meyer Test [56], the Arm Motor Ability Test [57], and the pain Visual Analogue Scale [58], were not included in this review as the aim was to explore and examine how effective medical devices are used as interventions for improving function (activity and participation) of the UL in adult stroke survivors.

**Inclusion criteria.**

- English-language articles
- Studies recruiting people over the age of 18 years
- Studies evaluating upper limb wearable technology
- Studies reporting randomized controlled trials or randomized comparable trials
- Studies measuring activity and or participation as classified by the World Health Organization International Classification of Functioning, Disability, and Health
- Intervention that could be used independently by the stroke survivor
- Wearable and portable technology that measures or monitors activity
- Research article

**Exclusion criteria.**

- Non-English–language articles
- Studies recruiting people under the age of 18 years
- Studies not evaluating upper limb wearable technology
- Studies not reporting randomized controlled trials or randomized comparable trials
- Studies not measuring activity and or participation as classified by the World Health Organization International Classification of Functioning, Disability, and Health
- Intervention that could not be used independently by the stroke survivor
- Wearable and portable technology that does not measure or monitors activity
- Not a research article
- Studies where the intervention is not clearly defined (it was unclear to the authors that the study did or did not meet the inclusion/exclusion criteria)
- Study protocols
- Studies reporting a nonwearable, nonportable intervention

### Quality Assessment of Included Studies

The methodological quality of the included RCTs was assessed using the Cochrane Risk of Bias (CRoB) assessment criteria [59]. This addresses specific fields including sequence generation, allocation concealment, blinding of participants and personnel, blinding of outcome assessment, incomplete outcome data, and selective outcome reporting. RCTs were classed as having an overall low risk of bias if they were rated as *low* for 3 of the key areas: allocation concealment [60], blinding of outcome assessment, and completeness of outcome data. They were judged as overall high risk of bias if any of these key areas were judged as being an overall *high* risk. RCTs judged as being at an overall unclear risk of bias were so if any of the 3 areas above were judged as *unclear*.

For the included non-RCTs, the methodological quality was assessed using the Downs and Black Instrument [61]. This instrument provides a score for each study, and the maximum score is 37. It assesses the way studies report their findings, their external and internal validity as well as selection bias.

### Data Extraction

The titles, abstracts, and/or papers were screened by the authors LP and JP to find studies that meet the review inclusion criteria. Final papers were decided between the authors JP and LP, and any disagreement was resolved through discussion. A standardized Excel form was used to extract data and study characteristics. This is where information such as data on the interventions and participant characteristics were recorded. The author LP carried out the data extraction and checked for accuracy by the author JP. Whenever applicable, missing data were requested from the authors of the study.

### Outcome Measure Quality Assessment

When undertaking a systematic review, it is important to assess the quality of the outcome measures used in the included studies to ensure that the results are valid and reliable. To achieve this, 3 clear domains can be considered for each of the outcome measures used: (1) whether the psychometric properties of the scale have been assessed previously [62], (2) whether the clinimetric properties of the scale have been considered [63-67], specifically the Minimally Clinically Important Difference (MCID) [66], and (3) whether the statistical analysis of the data provided by the scale fulfills the requirements of measurement theory [68-70].

We identified all outcome measures (N=12) used in the 11 included studies and reviewed each individually to assess whether they fulfilled the first 2 domains outlined above. The outcome measures measuring activity included BBT [41]; ARAT [42]; BI [43]; CAHAI [44-47]; JTHFT [48]; WMFT [49]; MAL [50]; MAS [51]; RMA [53]; UEFT [54]; and QuickDASH [55]; and the outcome measures measuring participation included SIS [52].

This was determined by reviewing the literature of each of the outcome measures. How the outcome measure was used and how the data were scored and analyzed was then examined for each of the 11 included studies.

All 12 outcome measures were measures of activity (N=11) or participation (N=1) as classified by the WHO ICF [40].

### Data Synthesis

A narrative review is presented on the included studies with supporting evidence tables for study characteristics and findings, risk of bias, and outcome measure assessment. A meta-analysis was not undertaken because of the variability of outcome measures used across the 12 included studies.

### Appraisal of Evidence

Studies in this review include RCTs and randomized trials without a control group. The included studies were appraised using the levels of evidence [71]. This is to enhance the understanding of the best levels of evidence included in this review [72]. The highest level of evidence to this end is that of the RCT and is considered to be of *level 1* evidence. Randomized trials without a control group are considered to be of *level 2* evidence. This is important as the study design can affect the validity and reliability of results. For example, RCTs are often considered the *gold standard* of research evidence and the most reliable because of the measures taken to reduce the influences of confounds [73].

## Results

### Search Results

**Figure 1:**
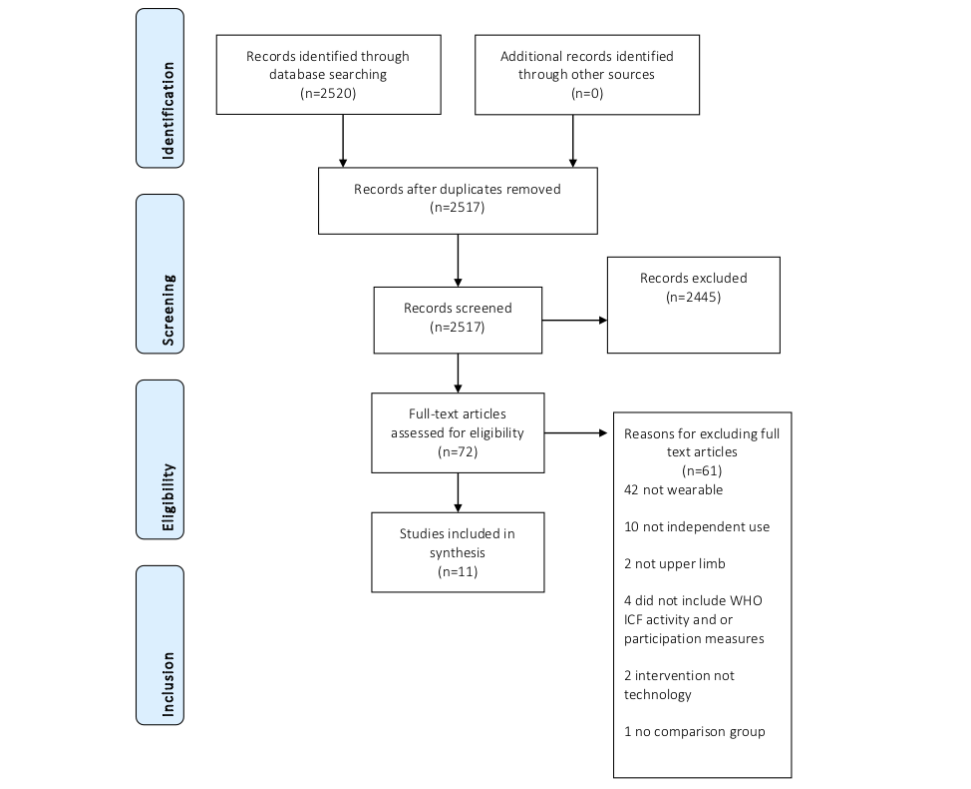
Article selection. WHO ICF: The World Health Organization International Classification of Functioning, Disability, and Health.

The electronic searches identified 2517 citations following deduplication. No additional citations were identified via handpicking methods. Following deduplication, 2517 records were screened and 2445 records were excluded through the title and abstract screening phases. At this stage, 72 full papers were obtained and 61 of these were excluded (reasons for exclusion can be found in Multimedia Appendix 2 [74-132]). Of these, 11 studies reported across 11 publications were included in this review (see Figure 1).

The review included 11 studies carried out in the United States (5), the Netherlands (2), Australia (1), Spain (1), Turkey (1), and Italy (1) with collectively 354 participants at baseline and 323 participants at final follow-up including control groups and participants from 17 days to 5-year poststroke. Of which, 7 studies were RCT level 1 and 4 were level 2 comparison trials and 6 of the 11 studies included acute stroke survivors. Participants' stroke type and severity varied from mild to severe. The interventions used FES (3), a hand device/glove (7), and arm worn garment (1). The duration of the intervention was from 3 to 12 weeks with varying intensity. Only 1 study found significant between-group differences for systems functioning and activity (*P*≤.02). The 11 included studies in this review had small sample sizes ranging from 5 to 99 participants at an average (mean) age of 57 years old.

### Quality Assessment

 The CRoB quality assessment summary can be found in Table 1, the Downs and Black quality assessment for non-RCT designs in Table 2 and the outcome measure quality assessment in Multimedia Appendix 3. Full details of the CRoB assessment can be found in Multimedia Appendix 4 and the Downs and Black quality assessment in Multimedia Appendix 5. First, 2 of the 7 included RCTs were judged as having an overall high risk of bias [73]. Of these, 1 of these RCTs was judged to be at high risk of having incomplete outcome data [141], 1 reported outcome assessment was not blinded [142], and 3 did not report blinding of participants and personnel [142-144]. Then, 4 RCTs were judged as having an overall unclear risk of bias [144-147], and 1 of the included RCTs was considered to be at an overall low risk of bias [143].

Non-RCT evidence was assessed using the Downs and Black Instrument, as they were studies that did not involve control groups. Overall, the 4 studies assessed using this instrument received high scores for reporting domains (items 1-10) and internal validity bias (items 14-20). Overall, they obtained lower scores for the external validity (items 11-13), selection bias (items 21-26), and power (item 27). The maximum score that could be obtained from this instrument is 32. Of the 4 studies assessed using this method, the highest score obtained was 21 [148] and the lowest was 14 [149].

**Table 1:** Cochrane risk of bias quality assessment summary.

Author, year	Random sequence generation	Allocation concealment	Blinding of participants and personnel	Blinding of outcome assessment	Incomplete outcome data	Selective reporting	Overall judgment
Alon, 2008 [145]	Unclear	Unclear	Unclear	Unclear	Unclear	Low risk	Unclear
da Silva Cameirão, 2011 [141]	Unclear	Unclear	Unclear	Low risk	High risk	Low risk	High risk
Lannin, 2016 [143]	Low risk	Low risk	High risk	Low risk	Low risk	High risk	Low risk
Nijenhuis, 2017 [142]	Low risk	Unclear	High risk	High risk	Low risk	Low risk	High risk
Vilafane, 2018 [147]	Low risk	Unclear	Low risk	Low risk	Low risk	Low risk	Unclear
Wolf, 2015 [144]	Low risk	Unclear	High risk	Low risk	Low risk	Low risk	Unclear
Nakipoglu Yuzer, 2017 [146]	Low risk	Unclear	Unclear	Unclear	Low risk	Low risk	Unclear

**Table 2:** Downs and Black quality assessment summary.

		Author, year			
		Barry, 2012 [150]	Friedman, 2014 [148]	Knutson, 2016 [151]	Prange-Lasonder 2017 [149]
**Reporting**					
	1^a^	1	1	1	1
	2^b^	1	1	1	1
	3^c^	1	1	1	1
	4^d^	1	1	1	1
	5^e^	2	2	2	2
	6^f^	1	1	1	1
	7^g^	1	1	1	0
	8^h^	0	0	1	0
	9^i^	1	0	1	0
	10^j^	1	1	1	1
**External validity**					
	11^k^	0	1	0	0
	12^l^	0	1	0	0
	13^m^	0 UTD^n^	0 UTD	0 UTD	0 UTD
**Internal validity—bias**					
	14^o^	0	1	0	0
	15^p^	1	1	1	0
	16^q^	1	1	1	1
	17^r^	0 N/A^s^	1	1	1
	18^t^	1	1	1	1
	19^u^	0 UTD	1	1	0 UTD
	20^v^	1	1	1	1
**Internal validity—confounding (section bias)**					
	21^w^	0 UTD	1	1	1
	22^x^	0 UTD	0 UTD	0 UTD	0 UTD
	23^y^	1	1	1	1
	24^z^	0 UTD	1	0 UTD	0 UTD
	25^aa^	0 UTD	0 UTD	0 UTD	0 UTD
	26^ab^	1	0 UTD	1	0 UTD
**Power**					
	27^ac^	0	0	0	0
Total		16	21	20	14

^a^1: Clarity of aims, objectives, and hypothesis.

^b^2: Clarity of main outcomes described.

^c^3: Clarity of participant characteristics.

^d^4: Clarity of intervention description.

^e^5: Clarity of distributions of principal confounders in each group.

^f^6: Are the main findings of the study clearly described?

^g^7: Are estimates of random variability in data for main outcomes clearly described?

^h^8: Have all adverse effects related to the intervention been reported?

^i^9: Have lost to follow-up participant characteristics been described?

^j^10: Have probability values for main outcomes been reported except from where *P*<.001?

^k^11: Were the participants asked to take part in the study representative of the entire population from which they were recruited?

^l^12: Were the participants prepared to take part in the study representative of the population from which they were recruited?

^m^13: Were the staff, places, and facilities where the participants were treated representative of the treatment that the majority of patients receive?

^n^UTD: unable to determine.

^o^14: Was there an attempt to blind participants?

^p^15: Was there an attempt to blind those measuring the main outcomes of the intervention?

^q^16: If any study results were based on data dredging, was this made clear?

^r^17: In trials and cohort studies, do the analysis adjust for different lengths of follow-up of participants, or in case-control studies, is the time period between the intervention and outcome the same for cases and controls?

^s^Not applicable.

^t^18: Were the statistical tests used to assess the main outcomes appropriate?

^u^19: Was intervention compliance reliable?

^v^20: Were the main outcome measures used accurate (valid and reliable)?

^w^21: Were the participants in different intervention groups (trials and cohort studies) or were the cases and controls (case-control studies) recruited from the same population?

^x^22: Were the participants in different intervention groups (trials and cohort studies) or were the cases and controls (case-control studies) recruited over the same period of time?

^y^23: Were participants randomized to the intervention groups?

^z^24: Was the randomized intervention assignment concealed from both participants and health care staff until recruitment was complete and irrevocable?

^aa^25: Was there adequate adjustment for confounding in the analysis from which the main findings were drawn?

^ab^26: Were losses to follow-up taken into account?

^ac^27: Did the study have sufficient power to detect a clinically important effect?

### Quality Assessment of Measurement Scales

All of the outcome measures used in the 11 studies had the psychometric properties of the scale assessed previously with only the floor and ceiling effect of the BBT, JTHFT, the CAHAI, QuickDASH, and the UEFT not studied. The MAL had no evidence of content validity or predictive validity. The WMFT had no evidence of predictive and content validity or responsiveness.

From a clinimetric perspective, all of the scales with the exception of the UL item of the MAS, UEFT, and the JTHFT had defined MCID in the literature. All data, parametric or nonparametric, were analyzed using appropriate statistical methods. This quality assessment of outcome measurements used provides some confidence in the evidence reported by each study (see Multimedia Appendix 3 for further details of the outcome measures of quality assessment and Multimedia Appendix 6 for a summary of the included studies in this review).

The 11 included studies in this review had small sample sizes ranging from 5 to 99 participants at an average (mean) age of 57 years.

## Discussion

### Principal Findings

This review set out to answer the question What is the effectiveness of UL wearable technology for improving activity and participation in adult stroke survivors?

Following exclusions, outcome measure assessment and quality assessment, 11 studies were included (see Multimedia Appendix 6). Of the 11 studies included, only one [141] found significant between-group differences using the CAHAI. However, this study was assessed as being high risk (see Table 1 and Multimedia Appendix 4) because of having >20% dropout rate. This study also included acute stroke survivors <19 days poststroke, which could mean that improvements are subject to acute natural improvement such as the spontaneous recovery following stroke [152]. Some improvements were found across all the studies included for both the control and intervention groups who all had an increase in rehabilitation dosage. This may suggest that a key mechanism for improvement is increasing the amount of rehabilitation carried out, which has been recognized in the national clinical guidelines for stroke [5]. However, further research is required to distinguish between the mechanism of dosage and intensity where dosage is the amount of rehabilitation activity and intensity is the amount of rehabilitation over time [153]. In other words, is it more effective to carry out more rehabilitation or is more effective to carry out more rehabilitation in a shorter period of time?

The 11 studies included in this review had small sample sizes ranging from 5 to 99 participants at an average (mean) age of 57 years old, whereas in England, Wales, and Northern Ireland, the average age for men to have a stroke is 74 and the average age for women to have a stroke is 80 [154]. It is also important to note that only one of the RCTs [143] was assessed as being low risk using the CRoB tool and of the non-RCTs [148-151], all obtained low scores on the Downs and Black instrument (14-21 out of 32), particularly for the external validity and selection bias domains. This suggests that the results may be difficult to generalize to the wider stroke population. However, quality appraisal is reliant on adequate reporting and some interventions may rely heavily on direct clinical input, which negates the ability to blind the participants and assessors.

A total of 12 outcome measures were used across the studies (11 functionals and 1 participation) to assess the effectiveness of the intervention in their respective ICF domains. A review of each of the outcome measures used to determine if the psychometric properties, the clinimetric properties, and the method of analysis were suitable revealed that all data had been analyzed appropriately. However, the UL item of the MAS, UEFT, and the JTHFT are yet to establish the MCID. This is important as it represents the smallest improvement considered worthwhile by a patient. The concept of an MCID is offered as the new standard for determining effectiveness of a given treatment and describing patient satisfaction in reference to that treatment [155]. Although some studies may reveal statistical significance, this may not mean that the intervention has made a meaningful difference to the functional capability of the stroke survivor.

An RCT methodology aims to control the conditions of each arm of a study to reduce bias [156]. Using technology to facilitate independent poststroke rehabilitation involves combining complex interventions with a complex condition. No two strokes are the same and no two contexts of adoption are the same. There are many complex nuances involved in using a device, independently often in the home environment. Therefore, using methodologies that account for these differences are important such as realist evaluation [157,158].

The use of technology to facilitate independent poststroke rehabilitation has the potential to motivate stroke survivors in that they are often interactive, fun to use, and engaging [12]. Furthermore, technologies have the potential to measure intervention outcomes over long periods of time that would normally be undetectable (ie, muscle activity in microvolts); provide formative, summative, and real-time feedback to the user in ways that could not be provided by a therapist (ie, the use of readily available graphics); and provide guidance and instruction out of clinical sessions. However, the lack of large, robust clinical trials can limit the uptake and acceptance of technological interventions in clinical practice. Indeed, since Moore's law published in 1965, which observed that the number of transistors on integrated circuits doubles approximately every 2 years [159], one of the difficulties is keeping up with the speed of new technologies against the time it takes to provide high-level clinical evidence. However, the recent publication of the National Institute for Health and Care Excellence Evidence Standards Framework for Digital Health Technologies (DHTs) sets out to describe standards for the evidence that should be available, or developed, for DHTs to demonstrate their value in the UK health and care system could speed up the uptake of DHTs [160].

The results of the included studies were not combined for a meta-analysis due to the varied types of data collected for the primary outcome measures. It would also be difficult to compare primary outcomes across the included studies accurately as there were a wide variety of functional and participation outcome measures used across the studies.

Future research could focus on adopting the principles and concept of technology use rather than on a specific device. Nonetheless, conventional research rigor is still required including robust methodologies that account for the complexity of use, larger sample sizes that reflect the population, valid, reliable measurement tools with MCID values, and importantly, the technology is suitable for the purpose of use.

### Conclusions

This review found that there is little evidence in the literature to support the use of wearable technologies to improve activity and participation for independent UL rehabilitation following a stroke. However, this may be because of small sample sizes and the limitations of using an RCT and randomized comparison trial methodology with a complex intervention and with a complex condition. The studies included in this review did highlight that improvements can be made when more rehabilitation is carried out, but the mechanisms of this are yet to be investigated fully. Future technologies may have the potential to measure outcomes, provide feedback, and engage users outside of clinical sessions. This could provide a platform for motivating stroke survivors to carry out more rehabilitation in the absence of a therapist, which could maximize recovery.

## Appendix

Multimedia Appendix 1 Medline search strategy.

Multimedia Appendix 2 Full text papers and reasons for exclusions.

Multimedia Appendix 3 Details of outcome measure quality assessment.

Multimedia Appendix 4 Details of quality assessment for included RCTs.

Multimedia Appendix 5 Details of quality assessment for non-RCT study designs.

Multimedia Appendix 6 Summary of included studies in this review.

## Abbreviations

ARAT: Action Research Arm Test
BBT: Box and Blocks Test
BI: Barthel Index
CAHAI: Chedoke Arm and Hand Activity Inventory
CRoB: Cochrane Risk of Bias
DHTs: Digital Health Technologies
FES: functional electrical stimulation
ICF: International Classification of Functioning, Disability, and Health
ICT: information and communication technology
JTHFT: Jebsen-Taylor Hand Function Test
MAL: Motor Activity Log
MAS: Motor Assessment Scale
MCID: minimally clinically important difference
MeSH: Medical Subject Headings
NIHR: National Institute for Health Research
QuickDASH: short version of disabilities of arm, shoulder, and hand
RCT: randomized controlled trial
RMA: Rivermead Motor Assessment
SIS: Stroke Impact Scale
UEFT: Upper Extremity Function Test
UL: upper limb
WHO: World Health Organization
WMFT: Wolf Motor Function Test
