# Supplementary material for: Effectiveness of Upper Limb Wearable Technology for Improving Activity and Participation in Adult Stroke Survivors: Systematic Review
Source: J Med Internet Res. 2020 Jan 8;22(1):e15981. doi: 10.2196/15981 (PMC6996755; doi:10.2196/15981)
Supplement: Multimedia Appendix 1 [file jmir_v22i1e15981_app1.docx]

## Multimedia appendix One: Medline search strategy

Please insert here.

1 stroke.mp. or exp Stroke/ (284840)

2 CVA.mp. or exp Cerebrovascular Disorders/ (346288)

3 cerebrovascular accident.mp. or Stroke/ (93192)

4 exp Cerebrovascular Disorders/ or poststroke.mp. (345723)

5 exp Brain Ischemia/ or brain isch$emi$.mp. (103201)

6 brain vasc$.mp. (1319)

7 cerebrovas$.mp. (130235)

8 exp Cerebral Hemorrhage/ or h$emorrhage.mp. or exp Hemorrhage/ (375464)

9 1 or 2 or 3 or 4 or 5 or 6 or 7 or 8 (821899)

10 upper limb.mp. or exp Upper Extremity/ (167876)

11 upper extremity.mp. or exp Upper Extremity/ (169632)

12 arm$.mp. or exp Arm/ (252629)

13 exp Shoulder Joint/ or exp Shoulder/ or shoulder$.mp. (75859)

14 exp Hand/ or hand$.mp. or exp Hand Joints/ (694186)

15 axilla$.mp. or exp Axilla/ (40479)

16 elbow$.mp. or exp Elbow Joint/ or exp Elbow/ (35998)

17 exp Forearm/ or forearm$.mp. (43236)

18 finger$.mp. or exp Fingers/ (161217)

19 wrist.mp. or exp Wrist/ or exp Wrist Joint/ (40982)

20 10 or 11 or 12 or 13 or 14 or 15 or 16 or 17 or 18 or 19 (1179751)

21 exp Rehabilitation/ or exp Exercise Therapy/ or Rehab$.mp. (496060)

22 Physio$.mp. (5125871)

23 exp Physical Therapy Modalities/ or physical therap$.mp. (156613)

24 physiatric.mp. or exp "Physical and Rehabilitation Medicine"/ (36359)

25 exp Exercise Movement Techniques/ or exercise$.mp. or exp Exercise/ (400405)

26 biofeedback.mp. (9742)

27 sensory feedback.mp. or exp Feedback, Sensory/ (4244)

28 train$.mp. (512080)

29 therap$.mp. (5713444)

30 treat$.mp. (5482281)

31 motor skill$.mp. or exp Motor Skills/ (29659)

32 motor re-learn.mp. (0)

33 re-educate.mp. (91)

34 recover$.mp. (643713)

35 enhance$.mp. (1307974)

36 promote$.mp. (738531)

37 support$.mp. (9419452)

38 function$.mp. (3589979)

39 activit$.mp. (3053043)

40 physical$.mp. (854666)

41 21 or 22 or 23 or 24 or 25 or 26 or 27 or 28 or 29 or 30 or 31 or 32 or 33 or 34 or 35 or 36 or 37 or 38 or 39 or 40 (18929488)

42 technolog$.mp. (483635)

43 exp Technology/ or technology.mp. (668417)

44 information technolog$.mp. (12438)

45 information technology.mp. or exp Information Technology/ (11160)

46 IT.mp. (4323075)

47 ICT.mp. (4761)

48 (information and communications technolog$).mp. [mp=title, abstract, original title, name of substance word, subject heading word, floating sub-heading word, keyword heading word, organism supplementary concept word, protocol supplementary concept word, rare disease supplementary concept word, unique identifier, synonyms] (429)

49 assistive technolog$.mp. (2091)

50 assistive technology.mp. or exp Self-Help Devices/ (11616)

51 telehealth.mp. or exp Telemedicine/ (26023)

52 telehealth.mp. (3910)

53 telecare.mp. (691)

54 telerehab$.mp. (746)

55 wear$.mp. (58121)

56 42 or 43 or 44 or 45 or 46 or 47 or 48 or 49 or 50 or 51 or 52 or 53 or 54 or 55 (5004478)

57 9 and 20 and 41 and 56 (8661)

58 limit 57 to english language (7606)

59 limit 58 to yr="2000 -Current" (6218)

60 limit 59 to "all adult (19 plus years)" (3290)

61 limit 60 to (clinical study or clinical trial, all or clinical trial or comparative study or controlled clinical trial or randomized controlled trial) (1023)

***************************
