# Supplementary material for: Effectiveness of Upper Limb Wearable Technology for Improving Activity and Participation in Adult Stroke Survivors: Systematic Review
Source: J Med Internet Res. 2020 Jan 8;22(1):e15981. doi: 10.2196/15981 (PMC6996755; doi:10.2196/15981)
Supplement: Multimedia Appendix 2 [file jmir_v22i1e15981_app2.docx]

| Reason for exclusion | Paper |
| --- | --- |
| Not wearable | (Ang et al, 2015) [74]  Bach (2005) [75]  Cameiro (2012) [76]  Carey (2002) [77]  Germanotta (2018) [78]  Hayward (2013) [79]  Hussain (2017) [80]  Kairy (2016) [81]  Kim (2016) [82]  Kim (2017) [83]  Kimberley (2004) [84]  Kiper (2014) [85]  Klamroth-Marganska (2014) [86]  Lee (2015) [87]  Lemmens (2014) [88]  Leonardis (2015) [89]  Li (2018) [90]  Linder (2013) [91]  Lo (2010) [92]  Lum (2002) [93]  Lum (2006) [94]  Masiero (2006) [95]  Masiero (2007) [96]  Masiero (2011) [97]  Masiero (2014) [98]  McCabe (2015) [99]  McNulty (2015) [100]  Metzger (2014) [101]  Orihuela-Espina (2016) [102]  Prange (2015) [103]  Piron (2009) [104]  Rand (2014) [105]  Sale (2014) [106]  Saposnik (2010) [107]  Saposnik (2016) [108]  Takahashi (2016) [109]  Timmermans (2014) [110]  Tomi (2017) [111]  Turkbey (2017) [112]  Vanoglio (2017) [113]  Volpe (2008) [114]  Wei (2011) [115] |
| Not independent use | Ang (2010) [116]  Barker (2017) [117]  Huang (2018) [118]  Hwang (2012) [119]  Kim (2013) [120]  Liao (2012) [121]  Page (2013) [122]  Sustano (2015) [123] |
| No comparison group | Kumar (2013) [124] |
| Not upper limb | Bergmann (2018) [125]  Cannell (2018) [126] |
| Did not include WHO ICF measures of activity and or participation | Cruz (2014) [127]  Lin (2018) [128]  Shimodozono (2014) [129]  Sullivan (2012) [130] |
| Intervention not technology | Curado (2015) [131]  Lee (2012) [132] |
