# Supplementary material for: Effectiveness of Upper Limb Wearable Technology for Improving Activity and Participation in Adult Stroke Survivors: Systematic Review
Source: J Med Internet Res. 2020 Jan 8;22(1):e15981. doi: 10.2196/15981 (PMC6996755; doi:10.2196/15981)
Supplement: Multimedia Appendix 3 [file jmir_v22i1e15981_app3.docx]

| **Activity and participation outcome measures** | **WHO ICF domain** | **Psychometric properties** | **Clinimetric properties** |
| --- | --- | --- | --- |
| Box and Blocks Test | Activity | Not studied: floor/ceiling effect. Studied: Reliability (test/retest and inter-rater), Validity (no gold standard for concurrent validity), predictive validity, construct validity, responsiveness. | MCID^a^ 5.5/7.8 [133] |
| Action Research Arm Test (ARAT^b^) | Activity | Evidence exists for floor and ceiling effects, reliability (internal consistency, inter-rater) validity (content, concurrent, construct), responsiveness. | MCID 12 and 17 points [134] |
| Barthel Index | Activity | Evidence exists for floor/ceiling effects, reliability (internal consistency, test-re test, inter-rater), validity (no evidence for content validity) concurrent validity, construct validity, convergent/discriminant validity, responsiveness. | MCID 1.85 points [135] |
| Wolf Motor Function Test (WMFT^c^) | Activity | Evidence exists for floor/ceiling effects, internal consistency, test-re test reliability, inter rater reliability, concurrent validity, construct validity. No evidence for predictive and content validity or responsiveness. | MCID 1.0 points: [134] |
| Upper limb items of Motor Assessment Scale (MAS^d^) | Activity | Evidence exists for floor/ceiling effects, test-retest reliability, inter-rater reliability, content validity, construct validity and responsiveness. No evidence for internal consistency (reliability), discriminant validity. | Unavailable |
| Upper extremity function test | Activity | Evidence for inter-rater reliability, test-retest reliability, predictive validity and responsiveness. No evidence for floor/ceiling effects, content validity, concurrent validity, specificity or known groups validity. | Unavailable |
| Rivermead Motor Assessment | Activity | Evidence for floor/ceiling effects, internal consistency, test-retest reliability, inter rater reliability, content validity, concurrent validity, construct validity, known groups validity and responsiveness. | MCID 3 points [136] |
| Motor Activity Log (MAL^e^) | Activity and participation | Evidence for floor/ceiling effects, internal consistency, test-retest reliability, inter-rater reliability, concurrent validity, construct validity, convergent/discriminant validity, known groups validity, responsiveness, sensitivity and specificity. No evidence for content validity, predictive validity. | MCID 1.2 points [134] |
| Jebsen-Taylor hand function test | Activity | Evidence for internal consistency, content validity, responsiveness. No evidence for floor/ceiling effects. | Unavailable |
| Chedoke Arm and Hand Inventory | Activity | Evidence for internal consistency, test-retest reliability, inter-rater reliability, content validity, concurrent validity, construct validity, known groups validity and responsiveness. No evidence for floor/ceiling effects, predictive validity, | MCID 6.3 points [137] |
| Short version of disabilities or arm, shoulder and hand (QuickDASH^f^) | Activity, participation and systems function [138] | Evidence for internal consistency, content validity, construct validity. No evidence for floor/ceiling effects, internal consistency, inter-rater reliability, test-retest reliability, concurrent validity, predictive validity, known group validity, responsiveness, sensitivity and specificity. | MCID 12.85 points for QuickDASH [139] |
| Stroke Impact Scale | Participation | Evidence for floor/ceiling effects, internal consistency, test-retest reliability, content validity, concurrent validity, predictive validity, construct validity, known groups validity, convergent/discriminant validity, sensitivity and specificity and responsiveness. | MCID 16 [140] |
