# Supplementary material for: Effectiveness of Upper Limb Wearable Technology for Improving Activity and Participation in Adult Stroke Survivors: Systematic Review
Source: J Med Internet Res. 2020 Jan 8;22(1):e15981. doi: 10.2196/15981 (PMC6996755; doi:10.2196/15981)
Supplement: Multimedia Appendix 4 [file jmir_v22i1e15981_app4.docx]

## Multimedia appendix 4: Details of quality assessment for included RCTs

Across the included seven RCTs, two reported that randomisation was computer generated [143, 144], one reported a coin flipping method [146], one used simple randomisation [147] and one concealed envelopes [142]. These RCTs were judged as having a low risk for this domain. Two RCTs did not report their randomisation methods [141, 145] and were therefore judged as having an unclear risk for this domain. Six of the seven RCTs did not report their allocation process and were judged as having an unclear risk for this domain [141, 142, 144-147] and one reported their allocation process as blinded [143] and were judged as having a low risk of bias for this domain. Three of the included RCTs did not report their participant and personnel blinding methods and were judged as having an unclear risk for this domain [141, 145, 146], three reported participants and personnel were not blinded and were judged at high risk for this domain [142-144] and one reported their RCT as double blinded and was therefore judged as having a low risk of bias for this domain [147]. Four of the included RCTs reported their outcome assessors as being blinded and were judged as having a low risk for this domain [141, 143, 144, 147]. Two RCTs did not report whether their outcome assessments were carried out by a blinded assessor and were judged as having an unclear risk for this domain [141, 146] and one reported their outcome assessor was not blinded and received a high risk of bias for this domain [142]. Five of the included RCTs reported <20% drop out rate and were considered a low risk of bias for this domain [142-144, 146, 147]. One of the RCTs did not state if they had any study withdrawals and were judged as having an unclear risk for this domain [145] and one RCT reported a 20% or higher (21.1%; n=4/19) drop-out rate and received a high risk judgement for this domain [141]. Finally, six of the seven RCTs were judged as having an overall low risk of bias for the selective reporting domain [141, 142, 144-147] as they reported all primary and secondary outcome measures and three of these reported a study protocol [142, 144, 147]. The remaining RCT did not report two of their outcome measures and was therefore judged as having a high risk of bias for this domain [143].

Therefore, two of the seven RCTs were judged as having an overall high risk of bias [141, 142]. One of these studies was judged as having an overall high risk of bias as they had a more than 20% drop out rate [141] and the remaining RCT was judged as having an overall high risk of bias because the outcome assessor was unblinded [142]. Four of the seven RCTs were judged as having an overall unclear risk of bias [144-47]. One study was judged as having an unclear risk of bias because they did not report on allocation concealment, blinding of the outcome assessor or completeness of data [145]. Two RCTs were judged as having an overall unclear risk of bias as they did not report on allocation concealment [144, 147] and one RCT was judged as having an overall unclear risk of bias because they did not report on allocation concealment or the blinding of the outcome assessor [146]. Lastly, one of the RCTs was judged as having an overall low risk of bias [143]. This RCT was judged as low risk for allocation concealment, blinding of the outcome assessor and completeness of data. However, this RCT was additionally judged as having a high risk of bias for the blinding of participants and personnel (it was a single blind RCT) and for the selective reporting domain.
