# Supplementary material for: Effectiveness of Upper Limb Wearable Technology for Improving Activity and Participation in Adult Stroke Survivors: Systematic Review
Source: J Med Internet Res. 2020 Jan 8;22(1):e15981. doi: 10.2196/15981 (PMC6996755; doi:10.2196/15981)
Supplement: Multimedia Appendix 5 [file jmir_v22i1e15981_app5.docx]

## Multimedia appendix 5: Details of quality assessment for non-RCT study designs

### Study reporting

All four of the studies included in this quality assessment clearly defined their study aims, outcome measures, participant characteristics, intervention, principle confounders and their study findings [148-149]. Three of the studies provided random estimates of the random variability of the data for the main outcomes [148, 150, 151] and one did not [149]. Three of the studies did not report adverse events or how they were measured [148-150] and of the studies did [151]. Two of the included studies did not report the characteristics of participants lost to follow up [148, 149] and two did [150, 151]. All four studies reported specific probability values [148-151].

### External validity

Participants from one of the studies [148] were representative of the entire population as they were recruited through local hospitals and support groups. The remaining three studies did not recruit their participants from places that represent the entire target population as one study adopted convenience sampling [150], one academic medical centre [151] or one rehabilitation centre [149]. As a result, three of the studies participants were not representative of the entire target population [149-151]. All four studies were scored “0 unable to determine (UTD)” for the domain requesting information about the staff and facilities being representative of the care the rest of the population receive [148-151].

### Internal validity bias

Study subjects were not blinded in three of the four studies [149-151] and one study blinded therapists and participants until they received their treatment when they inevitably became unblended [148]. Attempts were however made to blind the outcome assessors for three of the four studies [148, 150, 151] and not for the remaining study [149]. The four studies did not provide evidence for any unplanned analysis [148-151]. The time period between the intervention and follow up was the same for participants all four studies [148-151]. Statistical tests adopted across all four studies were also deemed appropriate [148-151]. Compliance with the intervention was reported as being reliable for two of the four studies [148, 151] and was unable to determine for the two remaining studies [149, 150]. The main outcome measures used for all four of the studies were valid and reliable measures [148-151].

### Internal validity selection bias

It was not possible to determine if all participants in each group came from the same place in one of the studies as convenience sampling was adopted [150]. Participants in the remaining three studies did all come from the same location [148, 149, 151]. It was also not possible to determine if participants in all four of the trials were recruited during the same time period [148-151]. Participants in all four of the studies were however randomised to their groups [148-151]. Randomisation concealment was not reported and therefore unable to determine across three of the four studies [149-151] and randomisation was concealed from participants and staff in the remaining study [148]. Analysis adjustments for confounding variables such as intention to treat was not reported in the four studies and therefore not possible to determine [148-151]. Losses to patient follow up during analysis were not reported across the four studies. For one study, the proportion lost to follow up was too small to affect the main findings [150], one study did not report a drop out rate [148] therefore it was not possible to determine if losses to participant drop out were accounted for. One study did adjust for missing values for their statistical analysis [151] and the remaining study did report a drop out rate but did not report if any adjustments were made during data analysis [149].

### Power

Due to low sample sizes reported across the four studies [148-151], they have all been judged as not having sufficient power to detect clinically important effects.
