# Supplementary material for: Effectiveness of Upper Limb Wearable Technology for Improving Activity and Participation in Adult Stroke Survivors: Systematic Review
Source: J Med Internet Res. 2020 Jan 8;22(1):e15981. doi: 10.2196/15981 (PMC6996755; doi:10.2196/15981)
Supplement: Multimedia Appendix 6 [file jmir_v22i1e15981_app6.docx]

| Author, year, country, study design, level of evidence | Number recruited (N) and final follow-up (n) overall and between groups | Gender, stroke hemisphere (L/R^a^), baseline stroke severity, mean time since stroke | Intervention and control descriptions and length/frequency of intervention | Activity and participation outcome measures | Activity and participation outcome results, summary, and reported *P* values |
| --- | --- | --- | --- | --- | --- |
| Alon, 2008 [145], the United States, parallel RCT^b^: participants within both fast recovery and slow recovery groups randomly assigned to task specific training (control) or task specific training with FES^c^ (intervention), nonblinded, block RCT, level 1 | N=26 | Control: 5M^d^, 8F^e^; intervention: 9M, 4F; control: 6R, 7L; intervention: 4R, 9L; not reported, inclusion criteria include the Fugl-Meyer score between 2 and 10; FES: 4.0 (SD 2.4 days); control: 3.9 (SD 2.6 days) | Intervention: exercise and FES; control group: exercises varied as were individually tailored: passive/active assistive/active: task specific, eg, grasping, holding, moving, placing objects to and away from body, and adjusted accordingly; control: 30 min twice daily, 5 days a week until hospital discharge, then 30 min twice daily independently. Intervention: as above and FES 10 min 5 days a week, 4 times daily, increasing by 5 min daily. 2 of the 4 sessions with exercise and 2 without. | Box and Blocks Test, Jebsen-Taylor Hand Function Test (both activities) | Box and Blocks: n=0 could move blocks at baseline, n=8 could transfer 10 or more blocks after FES and 3 control participants could transfer 5 or more blocks after exercise. Finding not significant (*P*=.058). 18 participants were required in each group for results to have sufficient power. Jebsen-Taylor: no significance between group differences (*P*=.12). |
| Barry, 2012 [150], the United States, randomized comparison trial, level 2 | N22 (n)19. WHO^f^ therapy group: N11 (n)10; manually assisted therapy group N11 (n) 9 | WHO therapy group: 4M, 6F; 5R, 5L, 6 *moderate arm involvement*, 4 *severe arm involvement*, 4.1 years; manually assisted therapy group: 6M, 3F, 4R, 5L, 3 *moderate arm involvement* 6 *severe arm involvement*, 5.1 years. | SaeboFlex: wrist hand orthosis (WHO group); therapy with manual assistance from therapist (MAT^g^ group); 1 h a week for 6 weeks and home exercises prescribed to complete at home 4 times per week. | Action Research Arm Test, Box and Blocks (both activities), and Stroke Impact Scale (participation). | No between-group differences for any measure. Significant differences observed within the WHO group for ARAT (*P*=.04), significant within-group differences for MAT group for stroke impact scale (*P*=.03). |
| da Silva Cameira ̃o, 2011 [141], Spain, pilot RCT, level 1 | N19 (n) 15; N=13 (intervention); N=6 (control) | Intervention: 5M, 5F reported; control IOT: 3M, 2F; control NSG^h^ 1M, 3F; intervention: 3L, 7R; control IOT^i^: 2L, 3R; control NSG: 2L, 2R; Fugl-Meyer intervention: 37.9 (SD 12.1); control 24.4 (SD 11.4); intervention 11.5 days; control IOT: 16.8 days; control NSG: 13 days | Intervention: rehabilitation gaming system (RGS^j^); participant wears gloves with attached sensors and carries out actions to complete games viewed on a computer screen. Involved hitting, grasping, and placing; control: divided into 2 groups: intense occupational therapy (IOT): pure extended occupational therapy with emphasis on motor tasks similar to the ones promoted by the RGS, eg, object displacement, and object grasp. Nonspecific games (NSG) group: performed games with Nintendo Wii, involving movements with paretic arm that did not show any virtual body in response to their actions; 12 weeks | Barthel Index, Chedoke Arm and Hand Inventory (CAHI; both activities) | BI: no significant between group differences (*P*=.54). CAHI: significant between-group improvements reported (*P*<.05) |
| Friedman, 2014 [148], the United States, within-subjects randomized comparison trial: participants randomized to all of these groups in a different order, level 2 | N=12 | 7M, 5F, 4L, 8R; all participants demonstrated mild-to-moderate upper extremity impairment as defined by the upper extremity Fugl-Meyer score (range 34-62) and mild-to-severe hand impairment as defined by the upper extremity Box and Block test (range 1-55), 34.6(SD 32.5), range 8 to 63 (months) | Music glove: user wears a glove and selects notes that appear on a screen by making gripping movements to play a song. Correct notes displayed at the end of the game; conventional tabletop exercises, IsoTrainer (static gripping of static object); six 1-h treatment sessions 3 times a week for 2 weeks | Box and Blocks, ARAT, Wolf Motor Function Test (all activities) | Subjects benefited less from isometric therapy than MusicGlove training, but the difference was not significant (*P*>.09). Box and Blocks: improvement of 3.21 (SD 3.82) vs. −0.29 (SD 2.27) blocks; *P*=.010; ARAT and WMFT^k^ results not significant |
| Knutson, 2016 [151], the United States, 2-arm parallel group randomized comparison trial, level 2 | N80 (n) 64: control: N40 (n) 29; intervention: N40 (n) 35 | Control: 25M, 15F; intervention 26M, 14F, not reported (side effect is reported), Fugl-Meyer mean score: 32 control, 34 intervention; 26 moderately impaired (control); 25 moderately impaired (intervention), 1.6 years control, 1.8 years intervention | Used FES to assist in FTP; cNMES-assisted hand opening exercise at home plus sessions of FTP^L^ in laboratory; 12 weeks, 20 sessions twice a week (apart from assessment weeks), 10 sessions/week of self-administered repetitive hand opening exercise at home. Functional task practice (FTP) 60 min per session. | Box and Blocks Test: arm motor abilities test (all activities) | Box and Blocks Test: Participants with largest improvements were <2 years post stroke with moderate (not severe) baseline hand impairments. 6-month post treatment gains of the Contralaterally Controlled Functional Electrical Stimulation (CCFES) group, 9.6 (95% CI, 5.6-13.6), were greater than cNMES^m^ group, 4.1 (95% CI, 1.7-6.5), between-group difference of 5.5 (95% CI, 0.8-10.2), *P*=.023. Arm motor abilities test: no significant between-group differences. (*P*=.096) |
| Lannin, 2016 [143], Australia, feasibility pretest/posttest RCT, level 1 | N9 (n) 9: (n=5 intervention, n=4 control) | Control: 2M, 2F; intervention 5M, 0F; not reported (side effected is reported); not reported, inclusion criteria included score of <6 on item 6 or 7 of the MAS for stroke. Also, all had to meet Saebo splint minimum active movement criteria (ie, active range of motion of 10 degrees shoulder flexion/abduction and 10 degrees of elbow flexion/extension); control: 4.7 months; intervention 2.5 months | Intervention: treatment as usual, addition of SaeboFlex. Training included reach and grasp of soft foam ball, moving ball laterally to touch target, releasing ball into a crate. Control: treatment as usual, offered intervention at the end of study (wait-list control); 8 weeks (intervention 45 min 5 days a week over 8 weeks) | UL times from MAS^n^, Box and Blocks Test (both activities), Stroke Impact Scale (participation) | MAS: little change; BBT^o^; 3 improved (intervention), SIS^p^: stroke recovery item scores for 6 participants increased (3 in either group); 1 remained the same (control group); 1 participant’s score decreased (intervention group); data missing for 1 participant |
| Nijenhuis, 2017 [142], the Netherlands, RCT, level 1 | N20 (n) 19: control N10 (n)10; intervention N10 (n)9 | Intervention 7M, 2F; control 3M, 7F, not reported (side effected is reported), Fugl-Meyer mean score 35 control, 44 intervention. Stroke severity mild/moderate/severe: control: 2/4/4; intervention 2/3/4, 12 months control, 11 months intervention | Self-administered home-based arm and hand training using passive dynamic wrist and hand orthosis combined with computerized gaming exercises; Self-administered home-based arm and hand training using a prescribed conventional exercise from an exercise book; 6 weeks (30 min per day, 6 days a week) | ARAT^q^, motor activity log (both activities), SIS (participation) | ARAT: improvements from pre-evaluation to post evaluation (*P*=.016) for control group; sustained at follow up (*P*=.01. No between-group differences. SIS: No between-group differences. MAL: no significant between-group differences. |
| Prange-Lasonder, 2017 [149], the Netherlands, longitudinal randomized comparison pilot trial, level 2 | N=5 (n)5: (n=3 in assistive support group and 2 in training support group) | 3M, 2F, not reported (side effected reported), 4 mild, 1 moderate (mean Fugl-Meyer score of 55), not reported. | Assistive support group using HiM^r^ system (ambulant and wearable) at home: glove worn with imbedded sensors to play virtual computer game; training support group used HiM in a clinical setting, visualize progress via automated reports; 180 min a week for 6 weeks (both groups) | Jebsen-Taylor Hand Function Test (activity) | Jebsen-Taylor Hand Function Test: difference on subtest *picking up small objects* significantly different (*P*≤0.04), other comparisons not significant. |
| Vilafane, 2018 [147], Italy, RCT, level 1 | N=32 (n)32; intervention: N16 (n)16; control N16 (n)16 | Control 10M, 6F; 8R, 8L; Modified Ashworth Scale mean 0.1, not reported, does report participants strokes were not more than 3 months prior; intervention 11M, 5F, 7R, 9L, modified Ashworth Scale mean 0.1, not reported, does report participants’ strokes were not more than 3 months prior. | Gloreha: robotic device in the shape of hand in the form of a glove, movements mimicked on a computer screen; Additional traditional rehabilitation including exercises such as assisted stretching, shoulder and arm exercises, and functional reaching tasks; 15 to 30-min treatments (both groups). | Short version of disabilities of arm, shoulder, and hand (QuickDASH^s^) and Barthel Index (both activities) | Reports both measures demonstrated a significant time factor (BI: *P*=.001, QuickDASH *P*=.001) |
| Wolf, 2015, [144] the United States, prospective single-blinded RCT, level 1 | N=99 (n) 92; control: N48 (n) 45; Intervention: N51 (n) 47 | Control 31M, 17F; control 25M, 26F, not reported (side effected is reported), Fugl-Meyer assessment means: control: 33.3; intervention: 34.1, 127.1 days control, 115.5 days intervention | Intervention: HAAPI: Hand mentor pro and Home exercise program; control: Home exercise program only; 3 h a day, 5 days a week for 8 weeks. | ARAT, Wolf Motor Function Test (both activities) | Both groups developed improvements across all upper extremity outcomes. However, no difference between groups in change and motor function over time. ARAT: *P*=.15; WMFT: *P*=.27 |
| Nakipoglu Yuzer, 2017 [146], Turkey, RCT, level 1 | N=30 | Control: 9M, 6F; intervention: 8M, 7F; not reported (side effected is reported); not reported, inclusion criteria did include at least a stage 2 wrist spasticity according to the modified Ashworth scale (MAS), not reported, *duration of illness* is reported. | Intervention: FES. Stimulus intensity was increased to the level that could be tolerated by the patient. Control: passive ROM exercises, stretching exercises, and a wrist hand static splint was also used; 30 min a day, 5 days a week (total of 20 sessions). | Rivermead Motor Assessment, Barthel Index, upper extremity function test (all activities) | BI: no between-group differences reported, but a statistically significant difference was found between discharge BI values in favor of the study group (*P*<.05). RMA^t^: no between-group differences reported, significant difference between the admission and discharge values for these parameters in intervention group (*P*<.05). Upper extremity function test: no significant between-group differences |

^a^L/R: left/right.

^b^RCT: randomized controlled trial.

^c^FES: functional electrical stimulation.

^d^M: male.

^e^F: female.

^f^WHO: wrist hand orthosis.

^g^MAT: manually assisted therapy

^h^NSG: nonspecific interactive games.

^i^IOT: intense occupational therapy.

^j^RGS: rehabilitation gaming system.

^k^WMFT: Wolf Motor Function Test.

^l^FTP: functional task practice.

^m^cNMES: cyclic neuromuscular electrical stimulation.

^n^MAS: Motor Assessment Scale.

^o^BBT: Box and Blocks Test.

^p^SIS: Stroke Impact Scale.

^q^ARAT: Action Research Arm Test.

^r^HiM: hand in mind.

^s^QuickDASH: short version of disabilities of arm, shoulder, and hand.

^t^RMA: Rivermead motor assessment.
